# Sox17 Regulates Liver Lipid Metabolism and Adaptation to Fasting

**DOI:** 10.1371/journal.pone.0104925

**Published:** 2014-08-20

**Authors:** Samuel Rommelaere, Virginie Millet, Thien-Phong Vu Manh, Thomas Gensollen, Pierre Andreoletti, Mustapha Cherkaoui-Malki, Christophe Bourges, Bertrand Escalière, Xin Du, Yu Xia, Jean Imbert, Bruce Beutler, Yoshiakira Kanai, Bernard Malissen, Marie Malissen, Anne Tailleux, Bart Staels, Franck Galland, Philippe Naquet

**Affiliations:** 1 Centre d’Immunologie de Marseille-Luminy (CIML), Aix-Marseille University, UM2, Marseille, France; 2 Institut National de la Santé et de la Recherche Médicale (INSERM), U1104, Marseille, France; 3 Centre National de la Recherche Scientifique (CNRS), UMR7280, Marseille, France; 4 Department of Veterinary Anatomy, University of Tokyo, Bunkyo-ku, Tokyo, Japan; 5 Institut National de la Santé et de la Recherche Médicale (INSERM), Aix-Marseille University, UMR_S 1090, TGML/TAGC, Marseille, France; 6 University of Texas Southwestern Medical Center, Center for Genetics of Host Defense, Dallas, Texas, United States of America; 7 Institut Pasteur de Lille, INSERM U1011, Université de Lille 2, EGID, Lille, France; 8 Université de Bourgogne, Laboratoire BioPeroxIL, EA7270, Dijon, France; INRA, France

## Abstract

Liver is a major regulator of lipid metabolism and adaptation to fasting, a process involving PPARalpha activation. We recently showed that the *Vnn1* gene is a PPARalpha target gene in liver and that release of the Vanin-1 pantetheinase in serum is a biomarker of PPARalpha activation. Here we set up a screen to identify new regulators of adaptation to fasting using the serum Vanin-1 as a marker of PPARalpha activation. Mutagenized mice were screened for low serum Vanin-1 expression. Functional interactions with PPARalpha were investigated by combining transcriptomic, biochemical and metabolic approaches. We characterized a new mutant mouse in which hepatic and serum expression of Vanin-1 is depressed. This mouse carries a mutation in the HMG domain of the Sox17 transcription factor. Mutant mice display a metabolic phenotype featuring lipid abnormalities and inefficient adaptation to fasting. Upon fasting, a fraction of the PPARα-driven transcriptional program is no longer induced and associated with impaired fatty acid oxidation. The transcriptional phenotype is partially observed in heterozygous Sox17+/− mice. In mutant mice, the fasting phenotype but not all transcriptomic signature is rescued by the administration of the PPARalpha agonist fenofibrate. These results identify a novel role for Sox17 in adult liver as a modulator of the metabolic adaptation to fasting.

## Introduction

Liver plays an essential role in lipid metabolism and homeostasis. Sensing and adaptation to metabolic needs involves the contribution of nuclear receptors such as peroxisomal proliferator-activated receptors (PPAR) which contribute more specifically to the control of lipid storage/catabolism and the detoxication of lipophilic xenobiotics. PPARα is a key organizer of the liver response to fasting, fatty acid catabolism and ketone metabolism [1]–[3]. Upon high fat diet feeding, PPARα contributes to liver homeostasis by limiting steatosis and improving the atherogenic lipoprotein profile associated with type 2 diabetes and the metabolic syndrome [4], [5]. In addition to metabolic effects, PPARα has been shown to regulate detoxication and inflammatory pathways, hence preventing tissue damage associated with lipid overload and oxidation [6]. At the genomic level, PPARα-RXR bind to degenerate response elements upstream of their target genes which can also be occupied in a mutually exclusive manner by LXR-RXR dimers [7]. This promiscuity suggests that other factors may modulate the activity of favored complexes on transcriptional “hot spots”. Interactions with other transcription factors have been documented with HNF4α or GATA-6 in the control of *Acot1* or *Glut4* gene expression respectively [8], [9]. Occupancy of PPRE sites is also regulated during the fed/fasting cycle depending on the expression of the HNF4α target gene *Hes6* which, through interaction with HNF4α co-represses PPARα target genes in fed states [10]. Given the complex regulation of PPARα activation and its importance in the control of dyslipidemia, this justifies the identification of novel regulators and effectors of its function.

We recently demonstrated that the *Vnn1* gene is a PPARα target gene in the liver [11]. Vnn1 is a regulator of tissue response to stress [12], [13] which could play a role in the effector programs controlled by PPARα activation. Furthermore we showed that the level of serum Vnn1 (sVnn1) is a reliable reporter of PPARα activity in hepatocytes [11]. To identify new regulators of liver adaptation to fasting and, possibly, PPARα-dependent transcription, we screened ENU (N-ethyl-N-nitrosourea)-mutagenized mice for serum pantetheinase deficiency. We identified the Seric and Hepatic Impaired VAnin mutant mouse (SHIVA) which carries a mutation in Sox17 altering its transcriptional potential. This defect impairs lipid metabolism and adaptation to fasting; the transcriptional signature associated with the Sox17 mutation encompasses genes involved in adaptation to fasting and in part regulated by PPARα. This study shows for the first time the contribution of Sox17 to liver metabolism in adult mouse.

## Materials and Methods

### Ethical statement

C57BL/6 and mutant (SHIVA abbreviated SHI) mice were bred (6 mice per cage), fed on normal chow diet and maintained under specific pathogen-free (SPF) conditions at the animal facility of the CIML. Microbiological controls are performed on a monthly basis at the level of the CIML facility on sentinel mice, cohoused with test mice. All experiments were done in accordance with French and European guidelines for animal care. Animal experimentation at CIML is controlled by the “Comité d’Ethique de Provence” under the agreement number 14 which specifically approved this study. The principal investigator of this study had obtained the authorization to experiment on live animals (Préfecture des Bouches du Rhône, number 13–70). The experimental work described in this article involves harvesting small samples of blood by retro-orbital puncture on anesthetized mice, or harvesting organs on euthanized mice by cervical dislocation. When this program was started, these procedures which do not induce animal suffering, did not require to obtain a specific authorization. The ENU procedure on which our program was connected, has been recently reported [14]. All experiments were performed on 8–12 week old female mice (cohorts of 4 to 6 mice per experimental condition). Control and experimental mice originated from the same housing room and were systematically age-, weight (25–30 grs)- and gender-matched. The PPARα deficient mice were backcrossed on the C57BL/6 background and kept at the Pasteur Institute in Lille. The Sox17+/− mice were maintained in Y. Kanai’s laboratory (University of Tokyo). The reduced fertility and viability of these mice limited our experimental investigations since it was impossible to obtain homogeneous cohorts of age matched female mice.

### Pantetheinase activity

Enzymatic substrates (pPNA and pAMC) and Vnn1 ELISA to detect pantetheinase activity and serum Vnn1 have been recently described [11].

### Histology

Tissue lysates and western blots were performed as described [13]. For histology techniques, HE and Oil red O staining were performed as described [1].

### Plasmids

The myc-tagged Sox17 and Sox9 and pVCAM1-luciferase plasmids were kindly provided by P Koopman, University of Queensland, Australia [15]. The luciferase construct downstream of pH 4×4 artificial promoter was provided by Y Kanai. The pVnn1-luciferase and Vnn1 plasmids were previously described [12], [16].

### Cells and luciferase reporter assays

The AML12 hepatocyte and COS7 cell lines were purchased from ATCC. COS7 or AML12 cells (10^5^/well) were transiently transfected with Sox17 and Sox9 expressing vectors (0.8 µg) using Lipofectamine 2000 (Life Technologies). Cells were harvested 48 h post transfection, and luminescence was detected using the Dual-Luciferase reporter assay (Promega). Flow cytometry analysis was performed on a FACS Canto II analyzer (Becton Dickinson).

### Quantitative, RT-PCR Transcriptome analysis, bioinformatics and statistical analysis

RNA was extracted from various organs using the TRIzol reagent (Life Technologies) and further purified using the RNeasy mini kit (Qiagen). cDNA and quantitative RT-PCR reactions were processed as described [13]. Quantifications were normalized to that of HPRT mRNA and analyzed using the Mann-Whitney test. Primer sequences are shown in Table S1 in File S1. Hybridization on Agilent chips was performed on the TGML platform (TAGC UMR_S 1090, Marseille, France) and raw expression values were quantile normalized under R (version 2.13.0) and Bioconductor. Bioinformatics analysis is described in supporting information. Raw data are accessible at GEO site (GSE38968). In all figures, error bars represent SEM. Statistical analysis was mostly performed using unpaired t-test or ANOVA when appropriate (GraphPad Prism software) unless indicated otherwise.

### Metabolic assays

Metabolic measurements were performed at the Clinique de la Souris (IGBMC, Strasbourg, France) or at the Pasteur Institute, Lille. In some cases, measurement of triglyceride levels was performed after separation of lipoproteins hence excluding the contribution of free glycerol. Statistical analysis was performed using the Mann-Whitney test. Fenofibrate (Sigma) was administered daily by oral gavage at the dose of 200 mg/kg in 200 µl of carboxymethylcellulose (CMC) for one week.

## Results

### A Sox 17 mutant mouse with reduced seric pantetheinase activity

**Figure 1:**
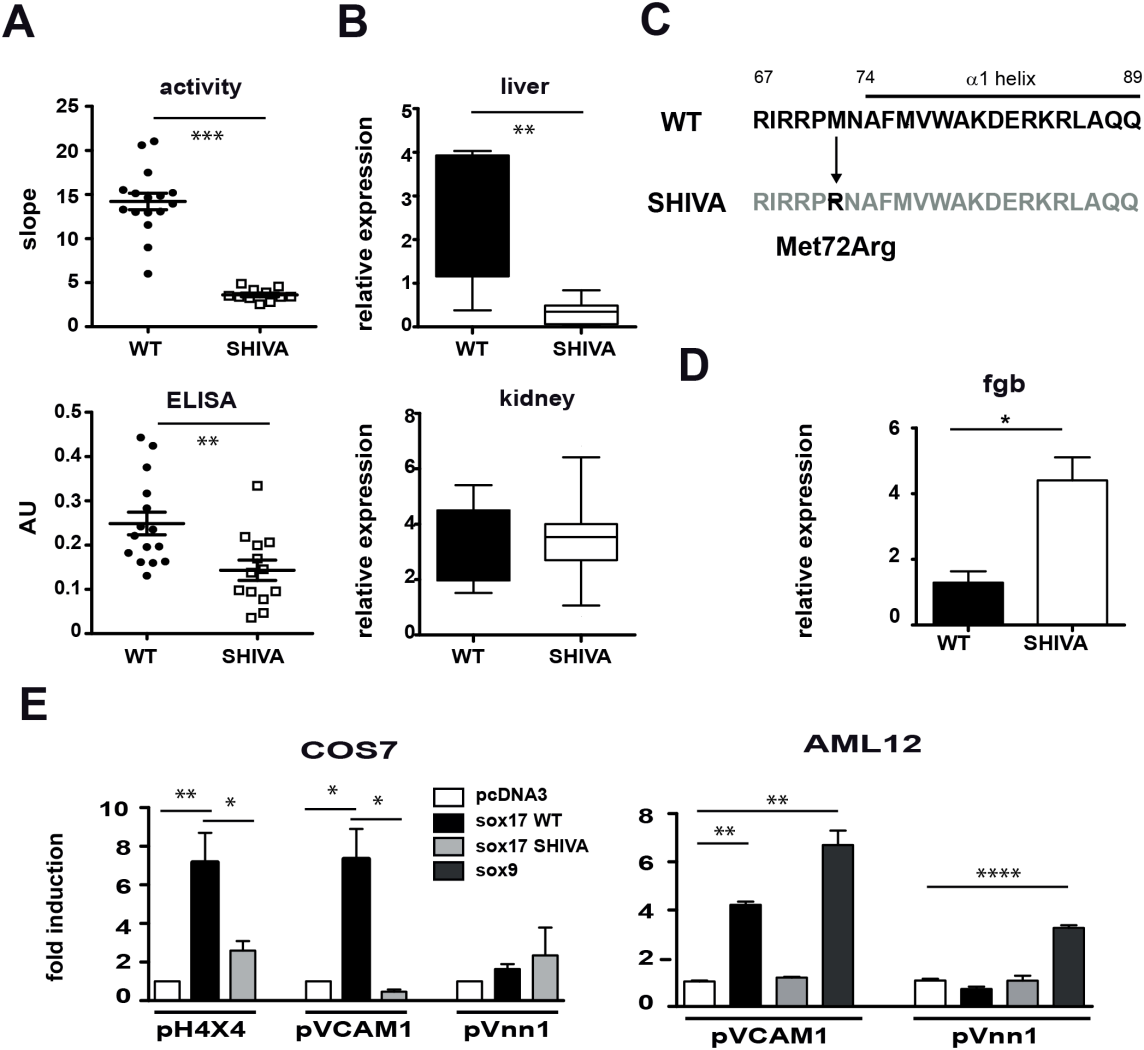
Sox17 mutation and transcriptional defect in the SHIVA mouse. Comparative analysis of Vnn1 expression in WT versus SHIVA mice using (A) Pantetheinase activity (pPNa substrate) and Vnn1 ELISA in serum (n = 13 to 16 mice); (B) qRT-PCR analysis of *vnn1* transcripts in liver and kidney (n = 4–5 mice); (C) Localization of the mutation in the HMG domain of the Sox17 protein. (D) Quantification by qRT-PCR of *Fgb* transcripts in liver from WT or SHIVA mice (*: p<0.05, **: p<0.005, ***: p<0.0005). (E) Luciferase reporter assays performed 48 hrs following cotransfection of the coding (Sox17, Sox9) and reporter (pH 4×4, pVCAM1, pVnn1) plasmids in COS7 or AML12 cells.

ENU-mutagenized G1 mice were screened for dominant mutations affecting sVnn1 production. As seen in Figure 1A, heterozygous SHIVA mice show reduced serum pantetheinase and sVnn1 levels. Quantification by qRT-PCR of *Vnn1* gene expression documents a specific reduction in liver but not in other Vnn1-expressing organs (Figure 1B). The expression level of the Vnn3 isoform is moderately reduced in liver but not spleen (Figure S1A in File S1). Chimeric wild type mice reconstituted with bone marrow from wild type or mutant SHIVA mice had normal levels of serum pantetheinase activity (Figure S1B in File S1), excluding a contribution of hematopoietic cells to the low pantetheinase phenotype of the SHIVA mouse. These and former [11] results show that the liver is the main source of serum Vnn1. The ENU SHIVA phenotype was detectable in heterozygous mice and segregated with the telomeric region of mouse chromosome 1 (LOD score >5). Coarse mapping co-localized it with marker D1Mit242 which is proximal to D1Mit169 in a 24 Mb critical region (Figure S2 in File S1). This region encompasses around 50 candidate genes among which *sox17* appeared as a likely candidate, since Sox9 was previously shown to transactivate *vnn1* gene expression in testis [15]. Sequencing of the *sox17* gene documented the presence of a T to G substitution, leading to a Met72 to Arg change (Figure 1C) in the DNA binding region of the alpha 1 helix of the HMG domain of the *sox17* gene [17]. Other mutations were excluded by sequencing of the telomeric end of chromosome 1 (supporting information data).

Sox17 expression in adult liver was further validated by immunohistochemistry (Figure S3A in File S1), qRT-PCR and immunoprecipitation (not shown). We verified that the SHIVA mutation did not impact the nuclear localization of Sox17 in transiently transfected COS7 cells (Figure S3B in File S1). Sox17 is described as a transcriptional repressor of the *Fgb* gene during development [18]. Results of qRT-PCR analysis (Figure 1D) indicate that *fgb* mRNA is 4–5 fold elevated in SHIVA mouse livers. We then tested the transactivating potential of the SHIVA Sox17 protein on reporter constructs *in* *vitro*. Luciferase reporter constructs containing either a tetramerized (pH 4×4) version of a prototypic Sox binding site, the *vcam1* promoter, a known Sox target gene [19] or the V*nn* promoters [12] were tested by transient transfection in COS7 or AML12 cells. As shown in Figure 1E, control Sox17 protein could activate the pH 4×4 and pVCAM1 promoters while the SHIVA Sox17 isoform was unable to induce luciferase expression. In contrast Sox9 but neither control nor SHIVA Sox17 could activate the *vnn1* promoter in the AML12 line. Altogether these results show that the SHIVA Sox17 protein is unable to activate or repress known target genes.

### Sox17 modulates PPARα-mediated transcription

**Figure 2:**
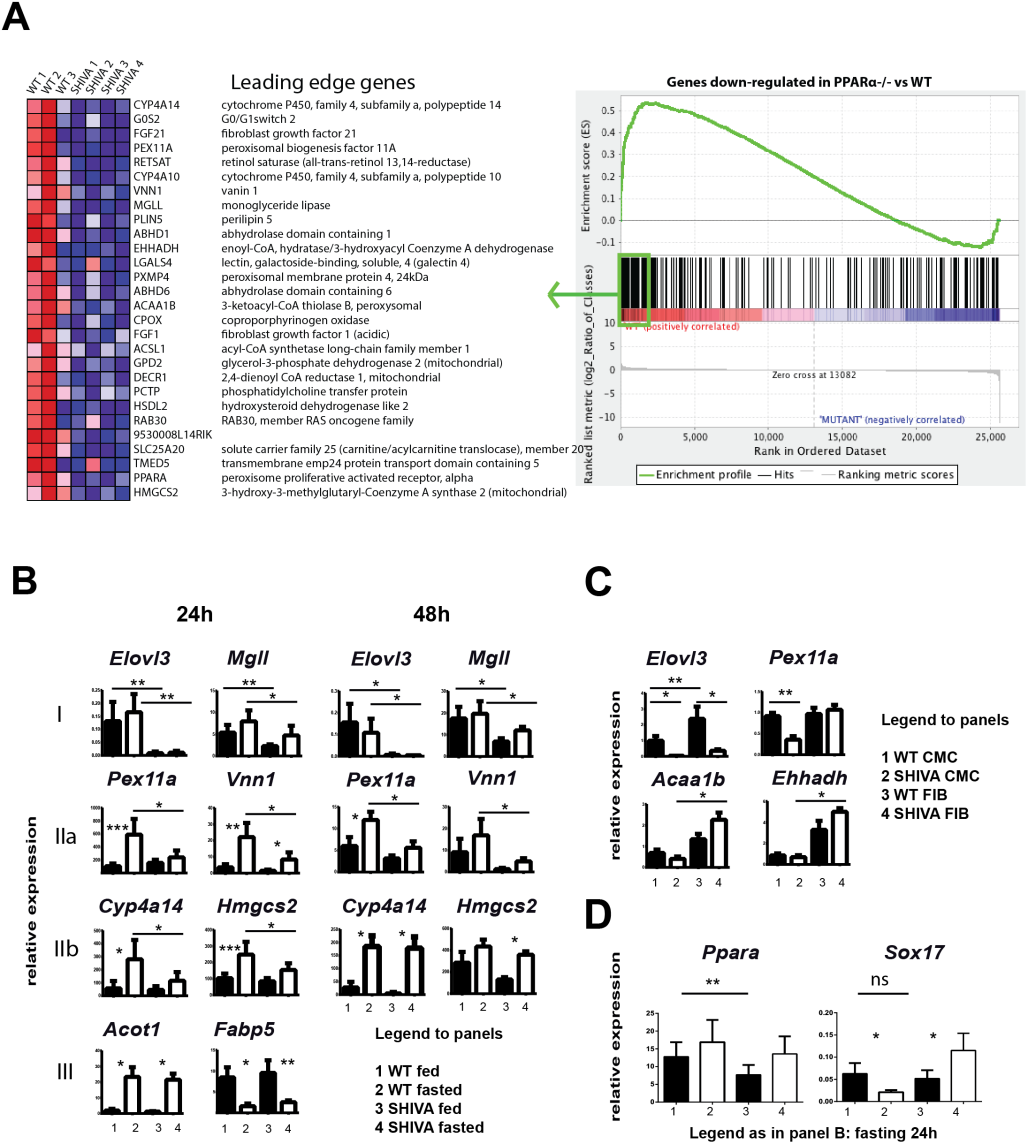
Altered PPARα-dependent transcription in SHIVA mice. GSEA comparing fasting SHIVA versus PPARα^−/−^ mice (n = 3–4 mice) (A) Heat map displaying the relative expression levels ranked from high (red) to low (blue) the most enriched genes (complete results are in Table S2 in File S1). Gene set enrichment plot showing skewing to the left, indicating enrichment in WT versus SHIVA livers of genes found down-regulated in PPARα-deficient mouse livers. Enrichment Score (ES): 0.5358231, Normalized Enrichment Score (NES): 2.257458, Nominal p-value: 0.0, FDR q-value: 0.04381425. (B) Quantification by qRT-PCR of various transcripts in livers from fed (black symbols) or 24 hours fasted (open symbols), WT versus SHIVA mice (n = 3 to 4 mice per condition). These mice are distinct from that used in the microarray analysis. (C) Similar analysis showing the relative expression evaluated by qRT-PCR performed on liver mRNA from 48 h fenofibrate-treated WT or SHIVA (SHI) mice for a series of PPARα target transcripts. (D) qRT-PCR analysis of *ppara* and *sox17* transcripts in fed or 48 hours fasted mice.

A preliminary transcriptomic analysis performed on fed SHIVA mice had documented an alteration of the transcription of few genes, including *Elovl3,* associated with fatty acid metabolism (not shown). Since Vnn1 transcription in the liver is strictly dependent on PPARα activation [11], we speculated that Sox17 might modulate transcriptional regulation by PPARα. We thus performed another transcriptomic analysis of wild type versus SHIVA livers under fasting conditions to induce PPARα activation. Transcriptional signatures highlighted a list of up and downregulated pathways (Table 1). Downregulated pathways are linked with lipid metabolism and fatty acid oxidation. Upregulated pathways are enriched in genes related to inflammation and to the metabolism of aminoacids or carbohydrates. We compared WT versus SHIVA expression data to all public genesets available from MSigDB (Broad Institute) using the GSEA algorithm. Briefly, the GSEA method allows to statistically test whether a set of genes of interest (referred to as a geneset) is distributed randomly in the list of genes that were pre-ranked according to their differential expression ratio between WT and SHIVA. We also re-analyzed public expression data (GSE8290) from fasted WT vs PPARα-deficient mouse livers in order to extract the genes that were found either up- or down-regulated and added them to the list of public genesets. As shown in Figure 2A (complete list in Table S2 in File S1), a significant fraction of genes found poorly induced in PPARα-deficient versus control fasted B6 mice were also found, as a group, significantly poorly expressed in fasted SHIVA mice (FDR q-value = 1.36E^−4^). To refine the analysis of the transcriptional patterns observed in the microarray, we performed qRT-PCR experiments on two independent cohorts of mice fed versus fasted for 24 h and 48 h (Figure 2B and Figure S4A in File S1). A first pattern (I) concerns genes whose transcription is either absent (*Elovl3*) or reduced (*Mgll, Cxcl14, Me1*) in both fed and fasted (24 and 48 h) SHIVA mice. A second pattern (II) concerns genes whose transcription is induced by fasting but this regulation is lost in 24 h fasted SHIVA mice. Among this group, the SHIVA phenotype is stable (IIa: *Vnn1, Pex11a, Pex1*) or reversible (IIb: *Cyp4a14, Hmgcs2, Acot4, Fabp2*) until 48 h fasting. Additional experiments using stimulation with fibrates abrogates most of the PPARα signature but the lack of Elovl3 expression is not affected (Figure 2C and Figure S4B in File S1). Some signatures are less robust and can vary between experiments (ie *Ehhadh*). A third heterogeneous group concerns the majority of transcripts which show similar behavior between fasted WT and SHIVA including some conventional PPARα target genes such as *acot1* or *fabp5*. Other transcripts related to inflammation (*Ccl2*), detoxication (*Gsta1*), or metabolic pathways (*Mvk*) show more heterogeneous patterns in SHIVA mice as previously seen in the microarray analysis (Figure S4 in File S1). Interestingly *Ppara* but not *Sox17* gene expression is reduced in fed SHIVA mice (Figure 2D). Also, Sox17 expression is downregulated by fasting in WT but not SHIVA mice. Another GSEA analysis underscored the downregulation of several genes associated with peroxisomal function in the SHIVA transcriptome (Figure S5 and Table S3 in File S1). In contrast, the expression of PPARα target genes [3] involved in mitochondrial fatty acid beta-oxidation (*Cpt, Acadm, Etfdh*) was induced by fasting in a comparable way between control and SHIVA livers (Figure S4 in File S1). Altogether, these results suggest that Sox17 might specifically control the expression of candidate target genes such as *Elovl3* and licenses a complete PPARα-induced transcriptional program [3].

**Table 1:** Pathway analysis of liver transcriptome in SHIVA mouse.

KEGG entry	pathway name	p-value
Downregulated pathways in SHIVA mouse		
mmu00071	Fatty acid metabolism	0
mmu00590	Arachidonic acid metabolism	0
mmu00830	Retinol metabolism	0
mmu01040	Biosynthesis of unsaturated fatty acids	0
mmu03320	PPAR signaling pathway	0
mmu00120	Primary bile acid biosynthesis	0.004
mmu00770	Pantothenate and CoA biosynthesis	0.008
mmu00561	Glycerolipid metabolism	0.02
mmu00982	Drug metabolism - cytochrome P450	0.027
mmu00930	Caprolactam degradation	0.039
mmu00100	Steroid biosynthesis	0.04
mmu00900	Terpenoid backbone biosynthesis	0.041
mmu00061	Fatty acid biosynthesis	0.042
**Upregulated pathways in SHIVA mouse**		
mmu00250	Alanine, aspartate and glutamate metabolism	0.001
mmu04640	Hematopoietic cell lineage	0.001
mmu04610	Complement and coagulation cascades	0.002
mmu00052	Galactose metabolism	0.004
mmu04612	Antigen processing and presentation	0.004
mmu04662	B cell receptor signaling pathway	0.004
mmu00010	Glycolysis/Gluconeogenesis	0.007
mmu00053	Ascorbate and aldarate metabolism	0.007
mmu00561	Glycerolipid metabolism	0.007
mmu00340	Histidine metabolism	0.008
mmu00620	Pyruvate metabolism	0.008
mmu00910	Nitrogen metabolism	0.008
mmu04630	Jak-STAT signaling pathway	0.008
mmu04910	Insulin signaling pathway	0.008
mmu00140	C21-Steroid hormone metabolism	0.009
mmu00410	beta-Alanine metabolism	0.009
mmu04620	Toll-like receptor signaling pathway	0.009
mmu04920	Adipocytokine signaling pathway	0.009
mmu00330	Arginine and proline metabolism	0.01
mmu00380	Tryptophan metabolism	0.01
mmu00480	Glutathione metabolism	0.01
mmu00720	Reductive carboxylate cycle (CO2)	0.01
mmu00310	Lysine degradation	0.013
mmu00982	Drug metabolism - cytochrome P450	0.016

### Sox17+/− mice display a SHIVA-like transcriptional profile

**Figure 3:**
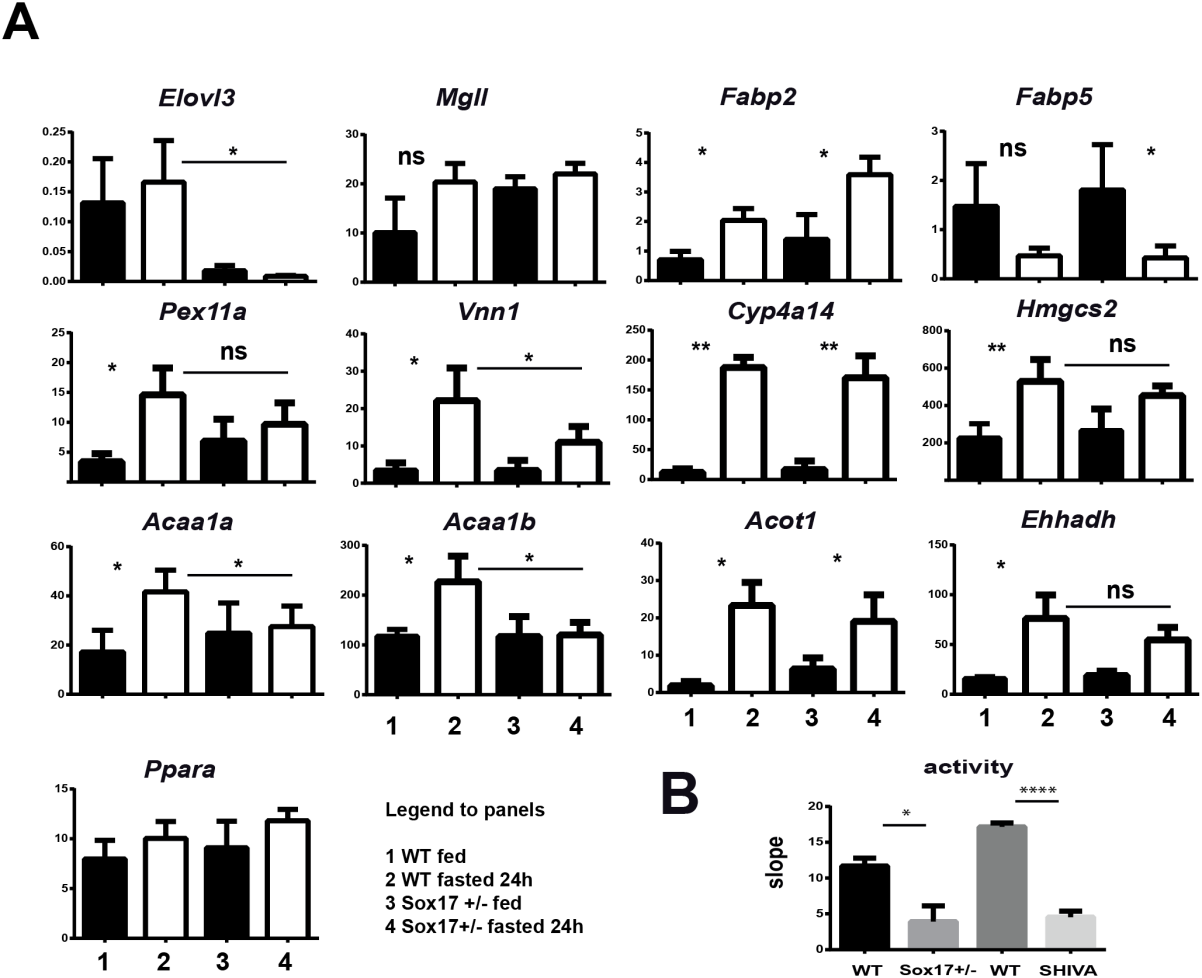
Transcriptional and functional defects in Sox17+/− mice. (A) Quantification by qRT-PCR of various transcripts in livers from fed (black symbols) or 24 hours fasted (open symbols), WT versus Sox17+/− mice (n = 3 to 4 mice per condition). (B) Quantification of sVnn1 and pantetheinase activity in the serum of control (WT), Sox17+/− or SHIVA mice (n = 3–4 per condition) all backcrossed on C57BL/6 mouse background (B6).

These transcriptional modifications could be directly or indirectly linked to the Sox17 mutation. To strengthen the functional link between the response to fasting and Sox17, we investigated the fasting response in fed versus fasted C57BL/6 Sox17+/− mice. We performed a qRT-PCR analysis on liver samples (Figure 3A) which again showed the strict dependency on Sox17 for the control of *Elovl3* expression. The results performed on fed versus 24 h fasted liver samples showed an alteration of fasting-mediated induction of *Vnn1, Acaa1b, Acaa1a* and to a lesser extent *Pex11a* transcripts. Intermediate responses were also observed for *Acot3, Acot4* and *Pex1* (data not shown) whereas no difference between WT and Sox17+/− mice was detected for other genes such as *Fabp2, Fabp5, Cyp4a14, Hmgcs2, Acot1, Ehhadh, Mgll and Ppara.* To confirm the contribution of Sox17 to the regulation of serum pantetheinase production, we analyzed the sera from Sox17+/− heterozygote mice. As shown in Figure 3B, the amount of Vnn1 pantetheinase activity is reduced in heterozygote mice kept on C57BL/6 mice to a level comparable to that observed in SHIVA mouse serum. Therefore, a Sox17 haploinsufficiency partially reacapitulates the SHIVA phenotype.

### Metabolic abnormalities in SHIVA mouse

**Figure 4:**
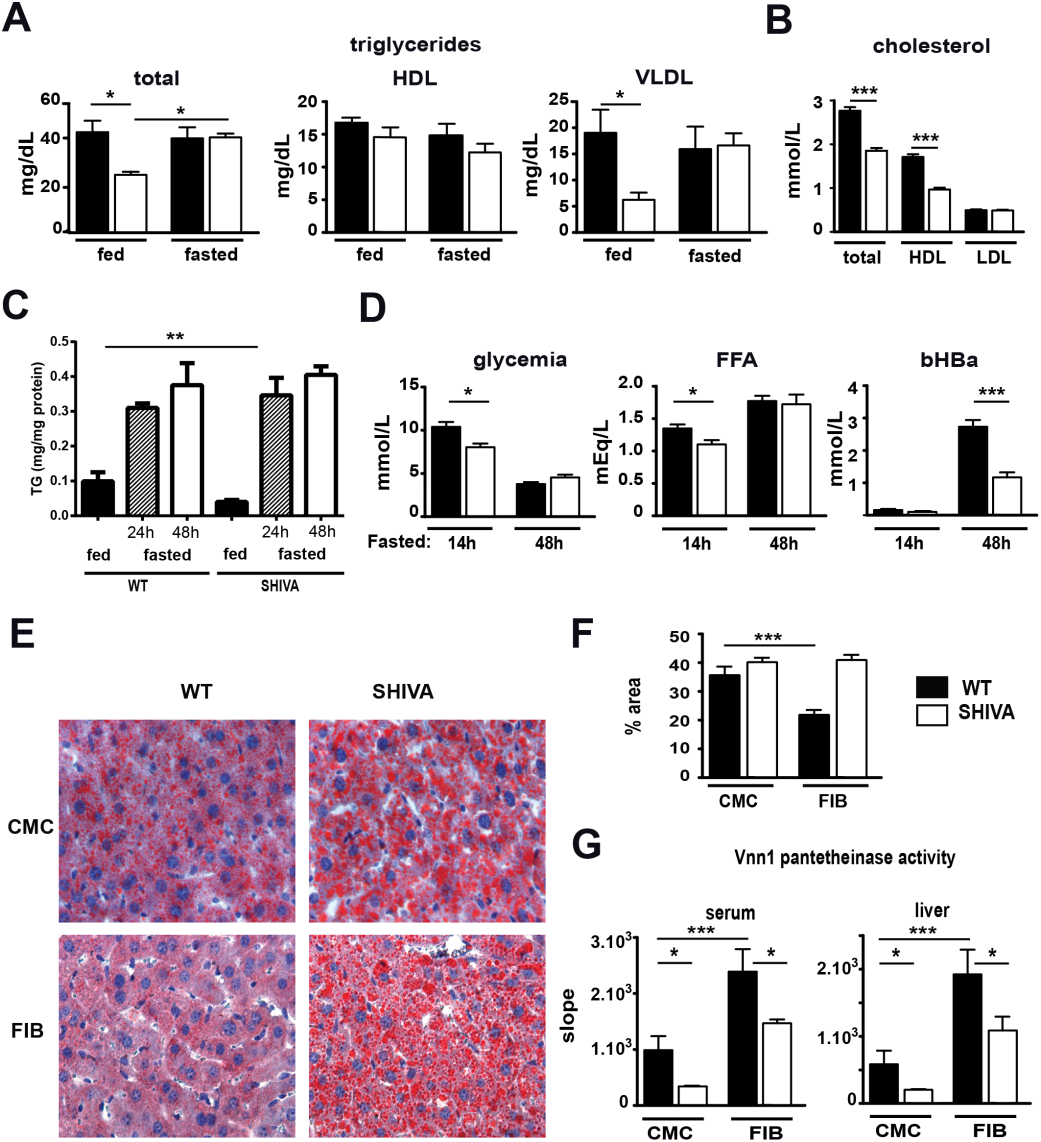
Impaired metabolic adaptation to fasting in SHIVA mouse. (A) Quantification of triglycerides in various lipoprotein fractions from fed or 16 hours fasted WT (filled bars) or SHIVA (clear bars) mice (n = 4 to 5 mice per condition). (B) Quantification of cholesterol in lipoprotein fractions (n = 5 to 8 mice per condition). (C) Quantification of triglycerides in liver from fed or 24/48 hours fasted mice (n = 4 mice per condition, representative experiment from 2 independent ones). (D) Glycemia, free fatty acids (FFA) and ketone bodies (beta hydroxyl butyric acid, bHBA) were measured after 14 h or 48 hours fasting. (E) Analysis of liver steatosis on oil red O stained OCT-frozen liver sections from carboxymethylcellulose (CMC) or fenofibrate (FIB)-treated-mice and (F) quantification using the ImageJ software by measuring the surface of the stained areas among 10 randomly-chosen fields per liver (3 mice per condition). Statistical analysis was performed using a two-way ANOVA test. (G) Pantetheinase activity (pAMC substrate) in serum or liver extracts from CMC (n = 3) versus FIB (n = 4) treated, WT or SHIVA mice.

Metabolic adaptation to fasting involves mobilization and degradation of fatty acids, a process regulated by PPARα in liver. PPARα-deficient mice develop hypertriglyceridemia and fasting-induced steatosis [1]. We performed metabolic measurements on fed versus fasted WT or SHIVA mice. In fed conditions, unlike PPARα-deficient mice, SHIVA mice had lower levels of triglycerides in the liver-derived VLDL (very low density lipoproteins) fraction (Figure 4A). After fasting triglyceride levels were comparable in WT and SHIVA mice. Furthermore, SHIVA mice showed lower HDL (high density lipoproteins) cholesterol levels (Figure 4B). A quantification of triglycerides in fed or fasted livers documented the presence of a lower level of triglycerides in fed SHIVA liver (Figure 4C). Fasting provoked a massive increase of TG induced by liver to a level and kinetics comparable between WT and SHIVA mice. The ability to cope with fasting-induced steatosis is controlled by the induction of lipid oxidation through PPARα activation. We thus evaluated the ability of SHIVA mice to control fasting-induced lipid overload in liver. We first analyzed the metabolic adaptation to a short versus prolonged fasting. WT and SHIVA mice mobilized free fatty acids and became severely hypoglycemic (Figure 4D). Importantly, control mice compensated the energetic demand by producing beta-hydroxybutyrate but this process was significantly reduced in SHIVA mice. Therefore SHIVA mice show a specific impairment in PPARα-dependent induction of fatty acid catabolism and/or ketogenesis. Given the preferential impact of Sox17 mutation on the expression of genes linked with peroxisomal proliferation such as Pex11a [20], [21] or enzymatic functions, we quantified the total amount of peroxisomes using a western blot analysis to probe PMP70, a structural membrane peroxisomal protein [22]. As shown in Figure S6 in File S1, we observed a two-fold reduction in liver peroxisomes in SHIVA mice compared to WT mice. We also quantified catalase activity as a global marker of peroxisomal function and Acox1 activity a rate limiting enzyme in peroxisomal beta-oxidation. In both cases fasting induced a reduction in activity as observed in rat [23]. However, no significant difference was observed between WT and SHIVA mice. Similar conclusions were obtained with acyl-CoA thioesterase activity using various acyl donors preferentially handled by Acot3 and 4 enzymes whose expression is partially impaired in SHIVA mice [24]. Therefore at the subcellular level, enzymatic activities between WT and SHIVA mice are comparable and defects might lie elsewhere.

Strong PPARα agonists reverse the transcriptional profile of fasted SHIVA mice. We quantified steatosis on liver sections stained with oil red O (Figure 4E, F) from WT and SHIVA mice treated with fenofibrate and fasted for 48 h. Fasted livers were enriched in lipid droplets in CMC-treated WT and SHIVA mice. Upon fenofibrate administration, the level of liver steatosis was lowered in control but not SHIVA mice. Similarly, using Vnn1 levels as an indicator of the efficiency of the ligand-induced PPARα response in liver and serum, we show that serum and Vnn1 pantetheinase activity was upregulated in WT and SHIVA mice but incompletely restored by fibrate administration in SHIVA mice (Figure 4G). Therefore, SHIVA mice combine evidence of impaired lipid metabolism under fed conditions and reduced adaptation to fasting which is partially rescued by using fenofibrate administration.

## Discussion

In this report, we identify Sox17 as a new regulator of lipid metabolism and adaptation to fasting in liver. Using sVnn1 as a reporter of liver PPARα activity [11], we isolated the SHIVA mutant mouse in which serum and hepatic pantetheinase activities are reduced. The SHIVA mouse harbors a mutation in the *Sox17* gene mapping to the HMG domain. This Met to Arg amino acid change is close to another Met which interacts with the DNA groove [17]. The mutant Sox17 molecule lost part of its ability to regulate target genes. Indeed, Sox17−/− mice are not viable due to a profound defect in definitive endoderm formation [25], whereas most heterozygous Sox17+/− C57BL/6 mice die before birth [26]. The few survivors display a low seric pantetheinase activity comparable to that found in SHIVA or PPARα deficient mice. In contrast, most heterozygous SHIVA mice survive and have a normal life expectancy. Although Sox17 participates to the development of pancreatobiliary progenitor [27] and endothelial [28] cells, SHIVA mice, albeit smaller in size at birth, show no major developmental defect at the adult stage with the exception of female hypofertility due to a partial atrophy of ovaries. Whether the Sox17 mutation impairs early developmental programs remains to be investigated.

The SHIVA mouse displays a metabolic and transcriptional phenotype involving lipid homeostasis and adaptation to fasting. More specifically, a defect in the induction of transcripts regulated by PPARα activation including *Vnn1* is documented. The metabolic phenotype of SHIVA mice combines a hypotriglyceridemia mostly in the VLDL fraction and in the liver and a reduced cholesterol in the HDL fraction. In contrast, upon fasting, SHIVA like PPARα-deficient mice are more hypoglycemic than control mice and significantly impaired in the production of ketone bodies. These latter abnormalities suggest an alteration of PPARα-induced transcriptional program. This is reflected by the GSEA analysis which outlines similarities between fasted PPARα-deficient and SHIVA mice at the transcriptional level. Interestingly, the level of the PPARα transcript is moderately reduced in fed SHIVA mice. However, since only a fraction of PPARα target genes show diminished induction upon fasting, it is unlikely that the reduction in PPARα transcription can by itself explain the complex phenotype observed in SHIVA mice. Furthermore, prolonged fasting or administration of strong PPARα agonists partially rescues the SHIVA fasting phenotype, arguing in favor of reduced level of PPARα activation state, preferentially detectable on a restricted set of target genes. The phenotype combining a hypotriglyceridemia and reduced HDL-cholesterol levels is reminiscent of that observed in a model of liver-specific fatty-acid synthase (FAS) deficiency [29]. This report suggests that products of the FAS reaction regulate glucose, lipid and cholesterol metabolism by serving as endogenous activators of distinct physiological pools of PPARα in adult liver. There are only few genes whose transcriptional level is strongly reduced in fed SHIVA mice. Among them, the *Elovl3* gene is virtually not expressed in SHIVA and Sox17+/− mice. This elongase participates to the synthesis of long fatty acids incorporated in liver triglycerides. Consequently, elovl3-deficient mice show a significant reduction in VLDL-triglyceride levels [30], [31]. Although the signature is less drastic, other dysregulated genes in the fed state include the monoglyceride lipase (*Mgll*) involved in the production of free fatty acids which can be used for energy production or synthetic reactions [32] and the malic enzyme 1 (*Me1*) which contributes to NADPH production required for fatty acid synthesis [33]. Detailed metabolomic studies will be required to investigate whether hepatic synthesis of VLCFA which would provide strong PPARα agonists and fuel to peroxisomal beta oxidation is impacted in SHIVA mice. In addition, SHIVA mice show reduced HDL-cholesterol levels which cannot be explained by an increase in cholesterol excretion (unpublished results, coll A. Groen) or by an altered expression of genes involved in cholesterol metabolism. Interestingly, a peroxisome deficiency limits the initial steps of cholesterol biosynthesis [34]. Although quantification of the peroxisomal *Pex1* and *Pex11a* transcripts and pmp70 protein could reflect a reduction in the global amount of peroxisomes, we did not detect any difference in catalase or acox1 activities, therefore excluding a major peroxisomal functional deficiency in SHIVA mice. This probably justifies that steatosis developing in fasted SHIVA mice is comparable to that of WT mice.

The mechanism involving Sox17 in this regulation is still unclear. Sox transcription factors can bind specific DNA sequences but their mode of action depends on requisite partners for target specificity [35]. Through their ability to bind the minor groove of DNA, they can induce conformational changes that bring distant proteins on gene promoters closer together allowing their interaction. The level of PPARα activation is regulated at the level of the availability of ligands but also at the transcriptional level through competition/synergy with other transcription factors and post transcriptional modifications [36]. A first possibility would be that Sox17 physically cooperates with PPARα in the induction of a subset of genes such as Vnn1. Although we could demonstrate a physical interaction by GST-pulldown in vitro (data not shown), we were not able to document this interaction in cells or liver and neither to show that cotransfection of PPARα and Sox17 could modulate the transcription of Vnn1 or elovl3 genes in model cell lines. CHIP sequencing and proteomic experiments will be required to clarify this issue and may help understanding why the most affected transcripts are enriched in peroxisomal and ketogenic transcripts. Nevertheless this signature although metabolically patent, does not translate in a detectable functional deficiency when individual enzymatic activities such as catalase, Acot or Acox1 are tested. One cannot exclude that a more global reduction in several genes collectively involved in the same function, as suggested by the GSEA analysis might result in a partial functional deficiency. This hypothesis is also supported by the fact that this transcriptional signature is easily reversed by exogenous PPARα agonists. Therefore an alternative hypothesis could be that endogenously produced PPARα ligands might be lacking as in the FAS deficiency model. In this view, one would predict that rather than regulating PPARα target genes, Sox17 might target other genes such as *Elovl3* which might be involved in the production of these endogenous ligands.

In conclusion, Sox17 is identified as a new regulator of lipid metabolism and liver adaptation to fasting, maybe through a functional cooperation with PPARα.

## Supporting Information

File S1 **Figure S1)** (A) Quantification of Vnn3 transcripts by qRT-PCR in liver and spleen of WT and SHIVA mice. (B) Pantetheinase activity (pAMC substrate) in serum from control mice reconstituted with bone marrow from WT or SHIVA mice. **Figure S2)** Mapping of the Sox17 mutation in (B6xC3H/HeN)F1 backcrosses as described in supporting information. **Figure S3)** Sox17 expression in liver and cells. (A) Immunohistochemistry analysis of Sox17 expression (FITC) on frozen liver sections (DAPI in white). (B) Immunostaining of Sox17 (red) in COS7 cells (DAPI in blue) following transfection with control or SHIVA Sox17 plasmids (x63). **Figure S4)** (A) qRT-PCR analysis on two independent cohorts of fed versus fasted control and SHIVA mice: Representative additional qRT-PCR experiments were performed on other transcripts to confirm the microarray analysis and extend the results shown in Figure 2B. Mice were fasted 24 h. Results are organized in broad categories based on their involvement in global functions (Abcd2 and 3 are peroxisomal proteins, some results are not shown to simplify the figure). (B) qRT-PCR on liver samples from 6 days fibrate-treated WT or SHIVA mice. **Figure S5)** A peroxisomal signature reduced in fasted SHIVA mice. **Figure S6)** Analysis of liver extracts from fed or fasted WT and SHIVA mice. (A) Quantification of the PMP70 protein by western blot on liver extracts (B) Quantification of catalase and Acox1 activities; (C) Quantification of acot activities using various acyl-CoA FA. **Table S1)** Oligonucleotides used for qRT-PCR in this study. **Table S2)** Leading edge of gene list from the GSEA comparing fasted SHIVA versus PPARα-deficient mice. **Table S3)** Leading edge of gene list from the GSEA comparing fasted SHIVA versus KEGG peroxisome geneset.
(DOC)
