# Supplementary material for: Sox17 Regulates Liver Lipid Metabolism and Adaptation to Fasting
Source: PLoS One. 2014 Aug 20;9(8):e104925. doi: 10.1371/journal.pone.0104925 (PMC4139292; doi:10.1371/journal.pone.0104925)
Supplement: File S1 — Figure S1) (A) Quantification of Vnn3 transcripts by qRT-PCR in liver and spleen of WT and SHIVA mice. (B) Pantetheinase activity (pAMC substrate) in serum from control mice reconstituted with bone marrow from WT or SHIVA mice. Figure S2) Mapping of the Sox17 mutation in (B6xC3H/HeN)F1 backcrosses as described in supporting information. Figure S3) Sox17 expression in liver and cells. (A) Immunohistochemistry analysis of Sox17 expression (FITC) on frozen liver sections (DAPI in white). (B) Immunostaining of Sox17 (red) in COS7 cells (DAPI in blue) following transfection with control or SHIVA Sox17 plasmids (x63). Figure S4) (A) qRT-PCR analysis on two independent cohorts of fed versus fasted control and SHIVA mice: Representative additional qRT-PCR experiments were performed on other transcripts to confirm the microarray analysis and extend the results shown in Figure 2B. Mice were fasted 24 h. Results are organized in broad categories based on their involvement in global functions (Abcd2 and 3 are peroxisomal proteins, some results are not shown to simplify the figure). (B) qRT-PCR on liver samples from 6 days fibrate-treated WT or SHIVA mice. Figure S5) A peroxisomal signature reduced in fasted SHIVA mice. Figure S6) Analysis of liver extracts from fed or fasted WT and SHIVA mice. (A) Quantification of the PMP70 protein by western blot on liver extracts (B) Quantification of catalase and Acox1 activities; (C) Quantification of acot activities using various acyl-CoA FA. Table S1) Oligonucleotides used for qRT-PCR in this study. Table S2) Leading edge of gene list from the GSEA comparing fasted SHIVA versus PPARα-deficient mice. Table S3) Leading edge of gene list from the GSEA comparing fasted SHIVA versus KEGG peroxisome geneset. (DOC) [file pone.0104925.s001.doc]

Supplemental information

ENU mutagenesis

ENU mutagenesis and genetic mapping of the mutation was performed according to the procedure described in the literature . After ENU mutagenesis of male breeders, we screened pantetheinase activity in the serum of mice born from the first generation (G1) between mutagenized male mice and C57Bl/6 female mice. G1 male mice carrying the phenotype were backcrossed on female C57Bl/6 and this procedure was repeated for more than 12 backcrosses with a stable transmission of the phenotype. Once the mutation was mapped to *sox17*, we designed a PCR assay on genomic DNA for more precise genotyping and confirmed that all viable mutant mice were heterozygous. Female SHIVA mice were sterile thus preventing the production of homozygous SHIVA mice. Therefore we maintained the colony by systematic backcrossing SHIVA male mice on wild type C57Bl/6 female mice. The seric pantetheinase low phenotype was stably transmitted to around 25% offsprings throughout more than 12 backcrosses. This incomplete transmission suggested either that the dominant mutation had partial penetrance or that some heterozygous SHIVA mice died before birth. This latter hypothesis is supported by the fact that the size of the F1 (mMUTxfB6) progenies was twice smaller on average than that of control C57Bl/6 breeding pairs and that newborn had a 15% reduction in weight which was partially compensated after 3 weeks (not shown). Therefore this mutation might affect fertility and/or fetal development. To map the mutation, C57Bl/6 SHIVA male mice were backcrossed on C3H/HeN mice and DNA from probands tested using a combination of SNPs scanning the entire mouse genome (Figure S2).

Targeted resequencing of chromosome 1 telomere and genetic variant identification

*To identify all genetic variations of the SHIVA mouse, a targeted resequencing of the telomeric region of mouse chromosome 1 was performed as described , adapted for a SOLiD 4 sequencer. Briefly, short fragment (~150 nt) paired-end libraries were prepared using focused acoustic fragm entation (Covaris S2) and according to the preparation guide from Life Technologies's (cms_081748.pdf). Two custom-designed Agilent SurePrint G3 Mouse DNA Capture 1x1M Arrays were used to cover the targeted 24 Mb telomeric region on chromosome 1 (mm9, chr1:1-24071946) and perform the library enrichment according to manufacturer's protocol #G4458-90000. Templated beads were prepared using the SOLiD EZ Bead System (EZ Bead E80) and sequenced according to standard Applied Biosystems Life Technologies protocols. The sequence reads were analyzed with the BioScope software suite (cms_4448431.pdf). SNPs and Small InDels were identified using the Genome Variant Analyzer pipeline developed at TGML Sequencing Platform (GeVarA, manuscript in preparation).*

Plasmid generation

The Sox17 plasmid containing the Met72 to Arg modification was generated using the Quickchange site-directed mutagenesis kit (Agilent) by introducing a T to G point mutation using the following primer ATCCGGCGGCCGAGGAACGCCTTTATGGT. The control or SHIVA mCherry-P2A-Flag-Sox17 constructs were generated by PCR amplification and cloned in the pLV-tetO plasmid obtained from Addgene.

Histological analysis and immunofluorescence, western blot analysis

For Sox17 immunostaining, liver sections were fixed in formalin for 10 minutes, saturated and permeabilized in 0.3% triton X100, BSA 2%, donkey serum 5%. Anti Sox17 antiserum was incubated for 1hr at RT and revealed using an Alexa 488 donkey anti rabbit Ig serum (Invitrogen). Immunofluorescence (IF) analysis was performed on COS cells transfected with Sox17. Cells were fixed 10 min in formaline and permeabilized 1 min with methanol at -20°C. After washes, cells were saturated 3 h in PBS-1%BSA-10% normal donkey serum. Sox17 (R & D) primary antibodies were incubated with cells in a humidity chamber overnight at 4°C and revealed with an Alexa546 donkey anti-goat sera (Life Technologies). The anti Sox17 rabbit anti serum was provided by Y Kanai. The rabbit anti PMP70 antibody was purchased from Abcam.

Transcriptome analysis

The gene expression dataset corresponding to fasted control versus SHIVA livers have been deposited on the GEO database. We generated GeneSets using public expression data downloaded from the Gene Expression Omnibus database. This gene expression dataset (GSE8290) comparing livers from WT vs PPARα deficient mice fed or fasted for 24h, was normalized by RMA and genes UP or DOWN regulated at least 2 folds in PPARα-deficient versus WT mice were added as GeneSets to the original collection 2 of the Molecular Signatures Database v3.0 (MSigDB) . Pathway scores were calculated using a method combining expression data and annotated pathway enrichment analyses as described . We used the Gene Set Enrichment Analysis (GSEA) method from the Massachusetts Institute of Technology to statistically test whether specific GeneSets were selectively enriched in the Sox17 SHIVA mouse versus control condition. The statistical significance of the analysis was evaluated by calculation of the false discovery rate (q value) based on 1,000 random permutations between all the GeneSets.

Enzymatic assays

ACOX1 activity was assayed by fluorimetric based assay adapted for plate reader (Infinite 200 Pro, Tecan). A reaction mixture (190 μl) contained 50 mM Tris buffer at pH 8, 0.75 mM homovanillic acid, 20 μg.ml^-1^ horseradish peroxidase, 0.02% Triton X-100 and 75 µM palmitoyl-CoA substrate. All reactions were started by the addition of 10 μl of crude extract.

Catalase activity was assayed by spectrophotometric assay adapted for plate reader (Infinite 200 Pro, Tecan). All reactions were started by the injection of a 25°C warmed reaction mixture (190 µl) contained 50 mM Tris buffer at pH 7.4 and 20 mM hydrogen peroxide in a plate well containing 10 µl of an appropriate dilution of the crude extract. The kinetic of hydrogen peroxide consumption is followed at 240 nm for 1 minute.

Peroxisomes were purified from mouse liver homogeneates using Peroxisome Isolation Kit (Sigma-Aldrich) according to manufacturer’s instructions. Purified peroxisomes were resuspended in Acot buffer (200mM potassium Chloride, 10 mM Hepes, pH 7.4) and immediately analyzed for Acot activity. Acot Activity was measured spectrophotometrically at 412 nm with 5,5-dithiobis(2-nitrobenzoic acid) (DTNB) as previously described . Briefly, a mixture of 50 μM Acyl-CoA (Sigma-Aldrich) supplemented with BSA to a molar ratio of BSA/Acyl-CoA of 1:4.5 was incubated with peroxisomal fraction in Acot buffer containing 0.05 mM DTNB. Activity was calculated using an *E*_412_= 13,600 m^-1^.cm^-1^.

Legends to supplemental figures

Figure S1: (A) Quantification of Vnn3 transcripts by qRT-PCR in liver and spleen of WT and SHIVA mice. (B) Pantetheinase activity (pAMC substrate) in serum from control mice reconstituted with bone marrow from WT or SHIVA mice.

Figure S2: Mapping of the Sox17 mutation in (B6xC3H/HeN)F1 backcrosses as described in Supplemental information.

Figure S3: Sox17 expression in liver and cells. (A) Immunohistochemistry analysis of Sox17 expression (FITC) on frozen liver sections (DAPI in white). (B) Immunostaining of Sox17 (red) in COS7 cells (DAPI in blue) following transfection with control or SHIVA Sox17 plasmids (x63).


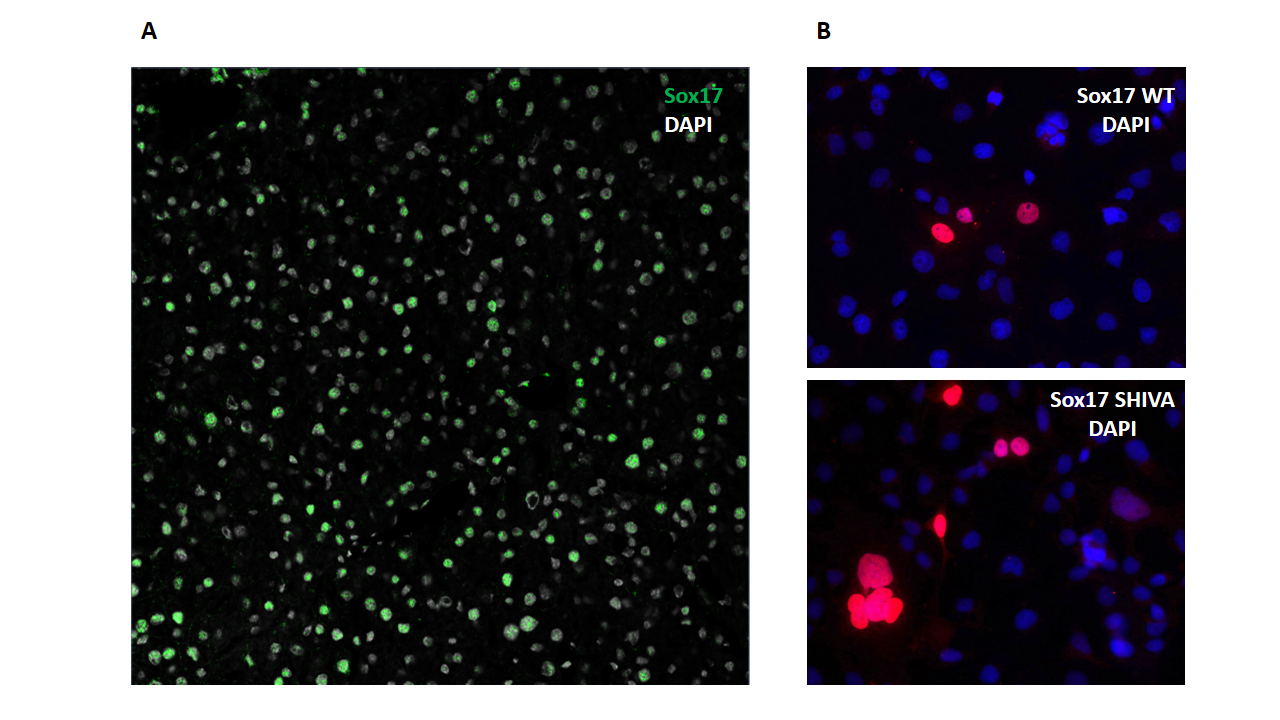


Figure S4: (A) qRT-PCR analysis on two independent cohorts of fed versus fasted control and SHIVA mice: Representative additional qRT-PCR experiments were performed on other transcripts to confirm the microarray analysis and extend the results shown in Figure 2B. Mice were fasted 24h. Results are organized in broad categories based on their involvement in global functions (Abcd2 and 3 are peroxisomal proteins, some results are not shown to simplify the figure). (B) qRT-PCR on liver samples from 6 days fibrate-treated WT or SHIVA mice.

**A**


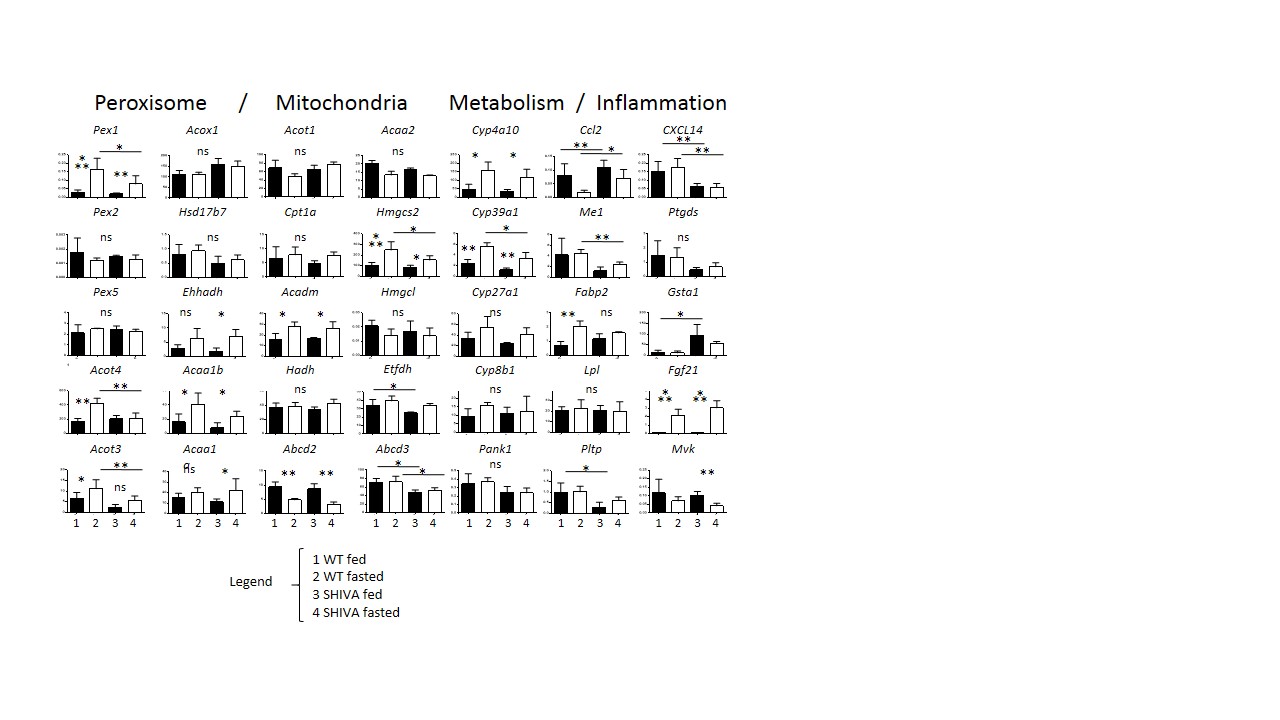


**B**

Figure S5: A peroxisomal signature reduced in fasted SHIVA mice.


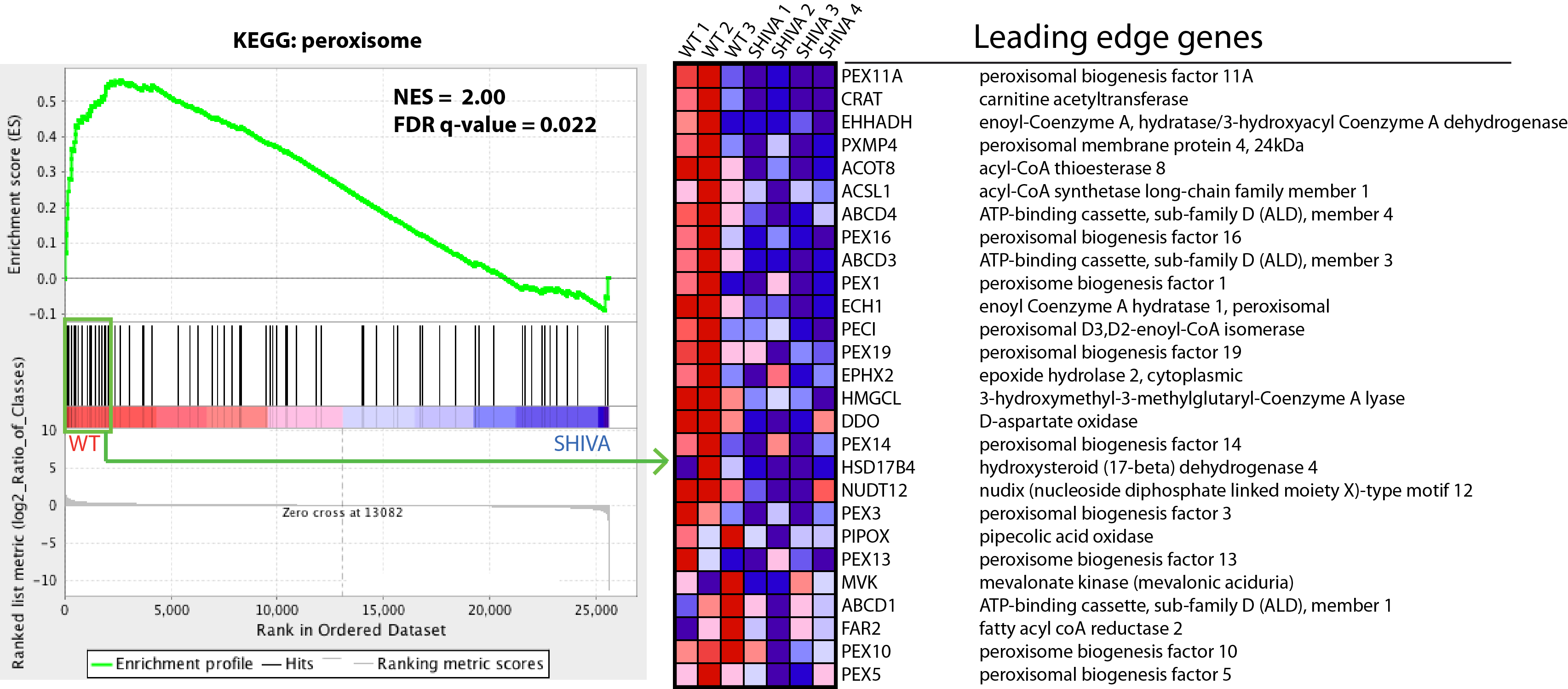


Pairwise comparison between 24h-fasted- WT vs SHIVA mice was performed using GSEA and the geneset collection 2 of the Molecular Signatures Database (MSigDB) from the Broad Institute. The geneset for the peroxisomal signature coming from the KEGG database (75 genes) was significantly (FDR q-values = 0.022) reduced in SHIVA versus WT livers, as indicated by the skewing of the geneset (i.e the bar code, where each vertical black line represents a gene of the geneset) towards the WT population (left panel), meaning that for a high proportion of the genes from this geneset, the expression level is higher in WT than in SHIVA mice. The relative expression levels of the 28 leading edge genes (genes that contribute the most to the observed enrichment) (green box) are displayed as a heatmap, from low (blue) to high (red) expression in WT vs SHIVA mice (right panel).

Figure S6: Analysis of liver extracts from fed or fasted WT and SHIVA mice. (A) Quantification of the PMP70 protein by western blot on liver extracts (B) Quantification of catalase and Acox1 activities; (C) Quantification of acot activities using various acyl-CoA FA.

Supplemental tables

Table S1: Oligonucleotides used for qRT-PCR in this study

|  | Forward | Reverse |
| --- | --- | --- |
| hprt | GCTTTCCCTGGTTAAGCAGTA | CAAACTTGTCTGGAATTTCAA |
| Sox17 | CGAGCCAAAGCGGAGTCTC | TGCCAAGGTCAACGCCTTC |
| Vnn1 | TGTGCGTTTCACCAGGGAT | ACTTGAGGGTCTGGGATCTCC |
| Vnn3 | TGTATGGAGTCCATCAAAGGCA | ACAAGATGTCTGAAAGCCGAATG |
| Pex11a | GACGCCTTCATCCGAGTCG | CGGCCTCTTTGTCAGCTTTAGA |
| Fgb | ACGATGAACCGACGGATAGC | CCGTAGGACACAACACTCCC |
| Acot1 | CCCCTGTGACTATCCTGAGAA | CAAACACTCACTACCCAACTG |
| Acot3 | GGAATTGGAAGTGGCCTTCTG | TCCATGTCCTTAGGGAGGTCC |
| Acot4 | AGCAGTGCGGTACATGCTTC | AGAGCCATTGATGGAAACTGTG |
| Acaa1b | caggacgtgaagctaaagcct | ctccgaagttatccccatagg |
| ehhadh | atggctgagtatctgaggctg | accgtatggtccaaactagctt |
| Elovl3 | ggacctgatgcaaccctatga | tccgcgttctcatgtaggtct |
| Fgf21 | CTGGGGGTCTACCAAGCATA | CACCCAGGATTTGAATGACC |
| Cyp4a10 | GTACATCTGTCACCTTCCCTGATGGACGCT | CAAACCTGGAAGGGTCAAACACCTCTGGAT |
| Cyp4a14 | CAAGACCCTCCAGCATTTCC | GAGCTCCTTGTCCTTCAGATGGT |
| Cyp39a1 | CAGTGTCCTGGAAGGTGGTT | GCTTTGGTAATGGGTCCAGA |
| p21 | GGACAGCAGAGGAAGACCAT | GAGTGGTAGAAATCTGTCATGCT |
| Fabp5 | CATCACGGTCAAAACCGAGAG | ACTCCACGATCATCTTCCCAT |
| CD74 | AGATGCGGATGGCTACTCC | TCATGTTGCCGTACTTGGTAAC |
| Cxcl14 | TACCCACACTGCGAGGAGAA | CGTTCCAGGCATTGTACCACT |
|  | Forward | Reverse |
| PPARa | CAGCAACAACCCGCCTTTT | GCAGTGGAAGAATCGGACCTC |
| Ptgds | GAAGGCGGCCTCAATCTCAC | CGTACTCGTCATAGTTGGCCT |
| Gsta1 | CCCCTTTCCCTCTGCTGAAG | TGCAGCTTCACTGAATCTTGAAAG |
| Mvk | GGTGTGGTCGGAACTTCCC | CCTTGAGCGGGTTGGAGAC |
| Mgll | CGGACTTCCAAGTTTTTGTCAGA | GCAGCCACTAGGATGGAGATG |
| Hmgcs2 | GAAGAGAGCGATGCAGGAAAC | GTCCACATATTGGGCTGGAAA |
| Abcd2 | ATACACATGCTAAATGCAGCAGC | GCCAATGATGGGATAGAGGGT |
| Pex1 | GACGCCTTCATCCGAGTCG | CGGCCTCTTTGTCAGCTTTAGA |
| Acox1 | TAACTTCCTCACTCGAAGCCA | AGTTCCATGACCCATCTCTGTC |
| Abcd3 | GGCCTGCACGGTAAGAAAAGT | CCGCAATAAGTAACAAGTAGCCT |
| Pex2 | TCCATGCCGCACTAGAGACTT | GAACCTGGTAGTACCGGAGGA |
| Hsd17b7 | TGGCAGAAGACGATGACCTC | GGCAGGATTCCAGCATTCAG |
| Cyp27a1 | AGGGCAAGTACCCAATAAGAG | TCGTTTAAGGCATCCGTGTAG |
| Pex5 | CTGGTGGAGGGCGAATGTG | GTCCTGGGTGAAATGGGTGG |
| Acaa1a | TCTCCAGGACGTGAGGCTAAA | CGCTCAGAAATTGGGCGATG |
| Cyp8b1 | CACGGGGATGTCTTCACGG | TGAGCACCAGTTCTTTTGCAT |
| Me1 | TCTGACTTCGACAGGTATCTC | CGGAATGCCAAACTGTACTGC |
| Acaa2 | CTGCTACGAGGTGTGTTCATC | AGCTCTGCATGACATTGCCC |
| Cpt1a | CTCCGCCTGAGCCATGAAG | CACCAGTGATGATGCCATTCT |

|  | Forward | Reverse |
| --- | --- | --- |
| Fabp2 | AAAGGAGCTGATTGCTGTCCG | CGCTTGGCCTCAACTCCTTC |
| Acadm | AGGGTTTAGTTTTGAGTTGACGG | CCCCGCTTTTGTCATATTCCG |
| Hmgcl | ATGGGGAATCTACCATCTGCT | AGGGAGTCCAGGTAACTGAGA |
| Hadh | TGCATTTGCCGCAGCTTTAC | GTTGGCCCAGATTTCGTTCA |
| Lpl | TTCCAGCCAGGATGCAACA | GGTCCACGTCTCCGAGTCC |
| Pltp | TGCTGAACATCTCCAACGCAT | CACTTTAATCCGACCACTGGAAT |
| Etfdh | GTGCGACTAACCAAGCTGTC | GGATGAACAGTGTAGTGAGTGG |
| Pank1 | ATGGTTGGATCGCTTGCTTGT | TCAGCCGCTGGGGTAAATTC |

Table S2: Leading edge of gene list from the GSEA comparing fasted SHIVA versus PPARα-deficient mice
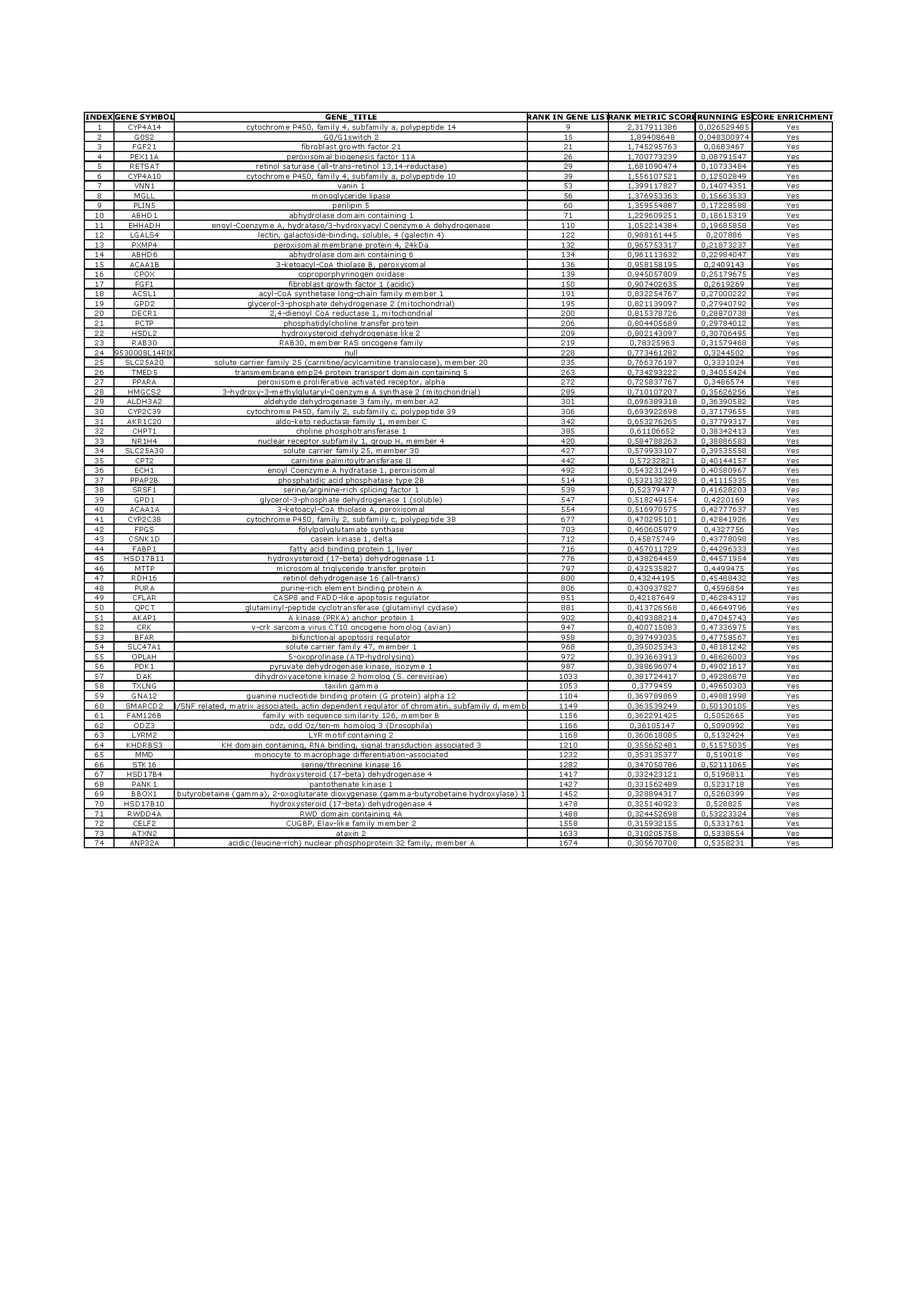


Table S3: Leading edge of gene list from the GSEA comparing fasted SHIVA versus KEGG peroxisome geneset


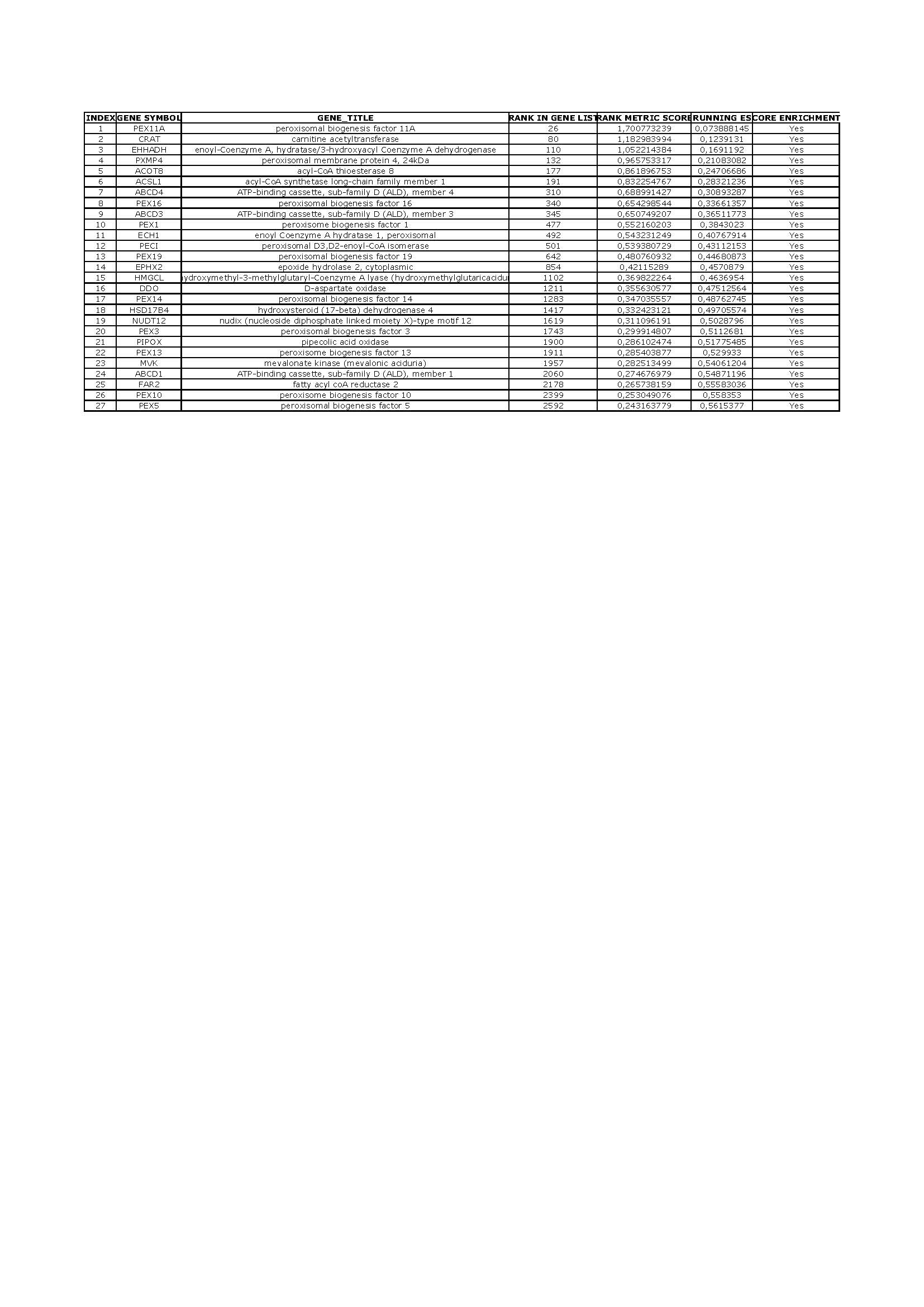


References to supplementary information

1. **Georgel P, Du X, Hoebe K, Beutler B.** 2008. ENU mutagenesis in mice. Methods Mol Biol **415:**1-16.

2. **Harakalova M, Mokry M, Hrdlickova B, Renkens I, Duran K, van Roekel H, Lansu N, van Roosmalen M, de Bruijn E, Nijman IJ, Kloosterman WP, Cuppen E.** 2011. Multiplexed array-based and in-solution genomic enrichment for flexible and cost-effective targeted next-generation sequencing. Nat Protoc **6:**1870-1886.

3. **Rakhshandehroo M, Sanderson LM, Matilainen M, Stienstra R, Carlberg C, de Groot PJ, Muller M, Kersten S.** 2007. Comprehensive analysis of PPARalpha-dependent regulation of hepatic lipid metabolism by expression profiling. PPAR Res **2007:**26839.

4. **Subramanian A, Tamayo P, Mootha VK, Mukherjee S, Ebert BL, Gillette MA, Paulovich A, Pomeroy SL, Golub TR, Lander ES, Mesirov JP.** 2005. Gene set enrichment analysis: a knowledge-based approach for interpreting genome-wide expression profiles. Proceedings of the National Academy of Sciences of the United States of America **102:**15545-15550.

5. **Majeti R, Becker MW, Tian Q, Lee TL, Yan X, Liu R, Chiang JH, Hood L, Clarke MF, Weissman IL.** 2009. Dysregulated gene expression networks in human acute myelogenous leukemia stem cells. Proceedings of the National Academy of Sciences of the United States of America **106:**3396-3401.

6. **Oaxaca-Castillo D, Andreoletti P, Vluggens A, Yu S, van Veldhoven PP, Reddy JK, Cherkaoui-Malki M.** 2007. Biochemical characterization of two functional human liver acyl-CoA oxidase isoforms 1a and 1b encoded by a single gene. Biochem Biophys Res Commun **360:**314-319.

7. **Jouve HM, Tessier S, Pelmont J.** 1983. Purification and properties of the Proteus mirabilis catalase. Canadian journal of biochemistry and cell biology = Revue canadienne de biochimie et biologie cellulaire **61:**8-14.

8. **Hunt MC, Solaas K, Kase BF, Alexson SE.** 2002. Characterization of an acyl-coA thioesterase that functions as a major regulator of peroxisomal lipid metabolism. The Journal of biological chemistry **277:**1128-1138.
